# Disruption of gap junctions attenuates acute myeloid leukemia chemoresistance induced by bone marrow mesenchymal stromal cells

**DOI:** 10.1038/s41388-019-1069-y

**Published:** 2019-10-24

**Authors:** Farah Kouzi, Kazem Zibara, Jerome Bourgeais, Frederic Picou, Nathalie Gallay, Julie Brossaud, Hassan Dakik, Benjamin Roux, Sophie Hamard, Louis-Romee Le Nail, Rita Hleihel, Amelie Foucault, Noemie Ravalet, Florence Rouleux-Bonnin, Fabrice Gouilleux, Frederic Mazurier, Marie C. Bene, Haidar Akl, Emmanuel Gyan, Jorge Domenech, Marwan El-Sabban, Olivier Herault

**Affiliations:** 1 CNRS ERL7001 LNOx “Leukemic Niche & Redox Metabolism”, Tours, France; 2 EA7501 GICC, University of Tours, Faculty of Medicine, Tours, France; 3 PRASE, DSST, Lebanese University, Beirut, Lebanon; 4 Biology Department, Faculty of Sciences, Lebanese University, Beirut, Lebanon; 5 Department of Biological Hematology, Tours University Hospital, Tours, France; 6 Department of Nuclear Medicine, Bordeaux University Hospital, Pessac, France; 7 Department of Surgical Orthopedia, Tours University Hospital, Tours, France; 8 Department of Anatomy, Cell Biology, and Physiological Sciences, Faculty of Medicine, American University of Beirut, Beirut, Lebanon; 9 Department of Internal Medicine, Faculty of Medicine, American University of Beirut, Beirut, Lebanon; 10 Department of Biological Hematology, Nantes University Hospital, CRCINA, Nantes, France; 11 Department of Hematology and Cell Therapy, Tours University Hospital, Tours, France

**Keywords:** Cancer microenvironment, Acute myeloid leukaemia

## Abstract

The bone marrow (BM) niche impacts the progression of acute myeloid leukemia (AML) by favoring the chemoresistance of AML cells. Intimate interactions between leukemic cells and BM mesenchymal stromal cells (BM-MSCs) play key roles in this process. Direct intercellular communications between hematopoietic cells and BM-MSCs involve connexins, components of gap junctions. We postulated that blocking gap junction assembly could modify cell–cell interactions in the leukemic niche and consequently the chemoresistance. The comparison of BM-MSCs from AML patients and healthy donors revealed a specific profile of connexins in BM-MSCs of the leukemic niche and the effects of carbenoxolone (CBX), a gap junction disruptor, were evaluated on AML cells. CBX presents an antileukemic effect without affecting normal BM-CD34^+^ progenitor cells. The proapoptotic effect of CBX on AML cells is in line with the extinction of energy metabolism. CBX acts synergistically with cytarabine (Ara-C) in vitro and in vivo. Coculture experiments of AML cells with BM-MSCs revealed that CBX neutralizes the protective effect of the niche against the Ara-C-induced apoptosis of leukemic cells. Altogether, these results suggest that CBX could be of therapeutic interest to reduce the chemoresistance favored by the leukemic niche, by targeting gap junctions, without affecting normal hematopoiesis.

## Introduction

The hematopoietic stem cell (HSC) niche is a specialized microenvironment of the bone marrow (BM) where HSCs reside, giving rise to all blood cells, and coexist with stromal cells. In hematological malignancies, it has been demonstrated that the BM microenvironment regulates leukemia development and progression. Acute myeloid leukemia (AML) is a heterogeneous clonal disorder characterized by the proliferation of BM blast cells with cytogenetic aberrations, recurrent somatic mutations, and alterations in gene expression [1, 2]. There is increasing evidence suggesting that the leukemic niche plays a critical role in the development and evolution of the disease, notably by promoting post-therapy chemoresistance. It has been recently established that leukemic cells present deregulated energy metabolism, and that BM niche supports leukemic cell metabolism pathways leading to leukemia chemoresistance [3, 4]. Like other tumor cells, leukemic cell metabolism is altered with an increase of glucose uptake known as Warburg effect [5]. AML cells present high glycolytic metabolism affecting cell proliferation and cell survival pathways [6, 7]. They show higher mitochondrial mass without concomitant increase in their oxidative phosphorylation (OXPHOS) [8–10].

Niche microenvironment regulates cell energetic metabolism by offering the needed nutrients [11]. Leukemic progenitors have close relationships with BM stromal cells either indirectly through secreted factors and released vesicles or directly via such cell–cell interactions as gap junctions [12]. Indeed, several studies have shown that secreted interleukins (i.e., IL-4, IL-10, and IL-13) affect the cell proliferation and apoptosis of treated AML leukemic cells, either by themselves [13] or when in coculture with endothelial cells [14], fibroblasts [15], or osteoblasts [16]. Intercellular communication with stromal cells has been shown to affect the function of AML cells [17], reducing both their proliferation and apoptosis [18] and increasing their drug resistance [19], thereby promoting AML relapse [20]. Besides, leukemic progenitors have been shown to have low level of reactive oxygen species (ROS) [21, 22]. Interestingly, connexins (Cxs), components of gap junctions, have been reported to be involved in the transfer of ROS toward the hematopoietic microenvironment [23]. This suggest that disruption of Cx-mediated interactions between AML cells and their niche could be a therapeutic target.

Cxs represent a family of transmembrane proteins which assemble into hemichannels formed by six identical or different Cxs, and one gap junction is composed of two hemichannels that allow the transfer of ions, miRNA, ATP, and other small metabolites between neighboring cells [24]. Cxs were found to be expressed in several types of tissues and tumor cells [25]. They are involved in cellular regulation by modulating intracellular pathways and gene transcription upon cell–cell contacts [26]. In leukemia, however, although some of them are expressed on blast cells, their role is poorly understood. Weber and Tykocinski have reported that direct contact of leukemic cell lines with clonal human KM-102 stromal cells decreases cell differentiation as a consequence of gap junction interactions [27]. Cx43 and Cx32 are expressed in OCIM2 and OCI-AML3 cell lines and the proliferative capacity of OCIM2 cells is related to the expression level of Cx43, suggesting its role in the regulation of cell proliferation [28]. Reikvam et al. have described the expression pattern of five different Cxs (Cx26, Cx32, Cx37, Cx43, and Cx45) in primary AML cells [29], and reported a high expression of Cx43 and Cx45, particularly in the most differentiated stages such as FAB M4 and M5) [30]. These studies support a role of Cxs as regulators of interactions between AML cells and their microenvironment.

Carbenoxolone (CBX) has been described for its role as a potent, effective, and water-soluble blocker of gap junctions [31, 32]. This glycyrrhetinic acid is a derivative from a natural triterpene compound [33]. In vitro studies have shown that CBX induces apoptosis and inhibits cell growth in various tumor cells, including breast and lung cancers [34, 35]. Moreover, CBX increases cell death in glioma cells expressing the tumor necrosis factor-related apoptosis-inducing ligand through inhibition of gap junctions [36]. In thyroid cancer cells, inhibition of intercellular communication by CBX decreases the phosphorylation of AKT and induces cell apoptosis [37].

Here, we identified a specific signature of Cx expression in BM-mesenchymal stromal cells (BM-MSCs) of the AML niche. Moreover, we show that CBX displays antileukemic activity through the extinction of energy metabolism, synergizes with cytarabine (Ara-C), and decreases the BM-MSCs-induced chemoresistance of leukemia cells to (Ara-C). In vivo experiments confirmed the antileukemic activity of CBX. The demonstrated lack of deleterious effects on normal BM-CD34^+^ hematopoietic progenitors suggests that CBX could be of clinical interest to improve the treatment of AML.

## Results

### Expression profile of Cxs in AML blast cells and BM-MSCs

**Fig. 1:**
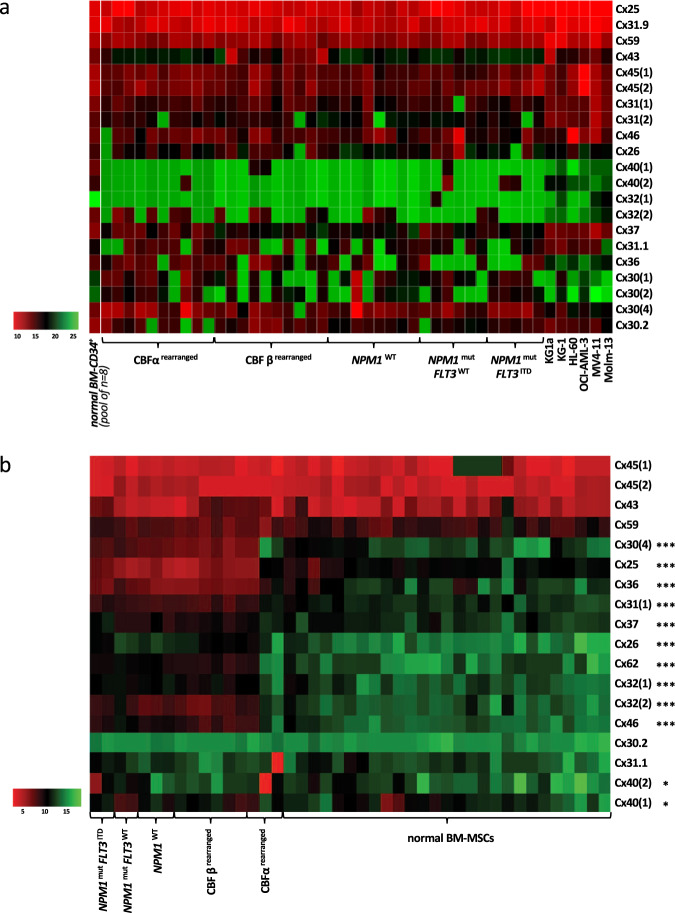
RNA signature of connexins (Cxs) in leukemic niche partners shows a specific expression profile in leukemic BM-MSCs. The expression of 23 Cxs was quantified by RT-qPCR (Supplementary Table 1). **a** The expression profile of detectable Cxs in 39 primary AML cells, six AML cell lines, and eight normal BM-CD34^+^ cells (pooled samples). Data were normalized to housekeeping genes and presented as ΔCt. **b** The expression profile of detectable Cxs in 19 primary AML BM-MSCs compared with 30 normal BM-MSCs. Data were normalized to reference genes and are presented as ΔCt. Heatmap colors reflect expression levels as ΔCt mean values (red: high expression, green: low expression). Comparative expression in BM-MSCs (leukemic BM-MSCs vs. normal BM-MSCs): *P < 0.05; **P < 0.01; ***P < 0.001

In order to investigate the expression of Cxs in AML cells, the mRNA expression of the Cx gene family (20 genes) was investigated in 39 different AML BM samples of different molecular/cytogenetic profiles and in six AML cell lines. qRT-PCR analyses revealed a high expression of three Cxs: Cx25, Cx31.9, and Cx59 in all AML primary samples with ΔCt values varying from 6 to 9 for Cx25, 7 to 10 for Cx31.9, and 7 to 10 for Cx59 (Fig. 1a). The expression of these three Cxs was not impacted by the cytogenetic nor molecular status of AML cells (CBFβ^rearranged^, CBFα^rearranged^, normal karyotype and *NPM1*^wt^, *NPM1*^mut^*FLT3*^wt^, or *NPM1*^mut^*FLT3*^ITD^). A similar expression pattern was observed in AML cell lines with ΔCt ranging from 6 to 10. Interestingly, Cx43 and Cx45 presented a low expression in all AML cells with ΔCt ranging from 9 to 16. Finally, two Cxs, Cx30(3) and Cx62, were not expressed in AML cells.

Normal BM-CD34^+^ similarly expressed high levels of Cx25, Cx31.9, and Cx59, but their expression was lower than in AML cells with ΔCt values varying from 8 to 11 for Cx25, 9 to 10 for Cx31.9, and 12 to 13 for Cx59. Normal BM-CD34^+^ cells were also characterized by an increased expression of Cx30, Cx36, and Cx40 comparatively to AML cells.

The expression of Cxs was quantified in 19 primary AML BM-MSCs samples and compared with primary normal BM-MSCs obtained from 31 healthy volunteers (Fig. 1b). In normal BM-MSCs, Cx45(1), Cx45(2), Cx43, and Cx59 were strongly expressed. A similar expression was observed in AML BM-MSCs, which also presented a specific significant overexpression of 12 Cxs: Cx25, Cx26, Cx30(4), Cx31(1), Cx32(1), Cx32(2), Cx36, Cx37, Cx40(1), Cx40(2), Cx46, and Cx62. Interestingly, among these Cxs, the most highly overexpressed was Cx25 with a 25-fold increase comparatively to normal BM-MSCs. Finally, Cx31.9, Cx30(1), Cx30(2), and Cx30(3) were not expressed in either types of BM-MSCs. The specific overexpression of various Cxs, especially Cx25, in BM-MSCs from AML samples, suggests a role for specialized gap junctions in the interactions between AML cells and their microenvironment which may constitute specific targets of the leukemic niche.

### CBX has antileukemic effect on AML cells without altering normal BM-CD34^+^ cells

**Fig. 2:**
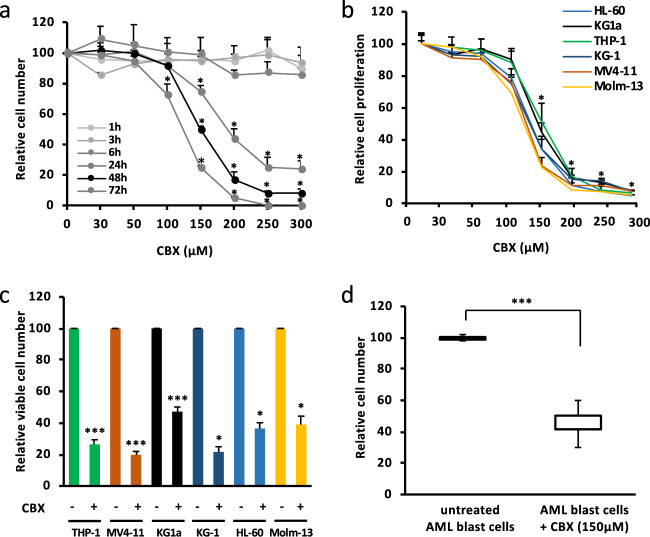
Carbenoxolone (CBX) reduces human AML cell proliferation. **a** CBX reduces KG1a cell proliferation in a dose- and time-dependent manner. A 50% decrease was observed after exposure to 150 µM of CBX for 48 h. The kinetics of CBX effect on the growth inhibition of KG1a cells, seeded at 2 × 10^5^ cells/mL, was estimated by trypan bleu exclusion assay (*n* = 5). **b** CBX exposure for 48 h significantly reduced leukemic cell growth of different AML cell lines (HL-60, KG1a, Molm-13, THP-1, KG-1, and MV4-11) with an IC_50_ ranging between 100 and 150 µM. AML cell proliferation was tested by MTT assay (*n* = 5). **c** CBX (150 µM, 48 h) reduced AML cell lines growth by at least 50%. Cell growth inhibition was determined by counting viable cells using trypan blue exclusion assay (*n* = 12). **d** CBX (150 µM, 48 h) reduced primary AML cell growth (*n* = 5). The number of viable cells after CBX exposure was expressed relatively to untreated cells. Results are expressed as mean ± SEM. *P < 0.05; ***P < 0.001

**Fig. 3:**
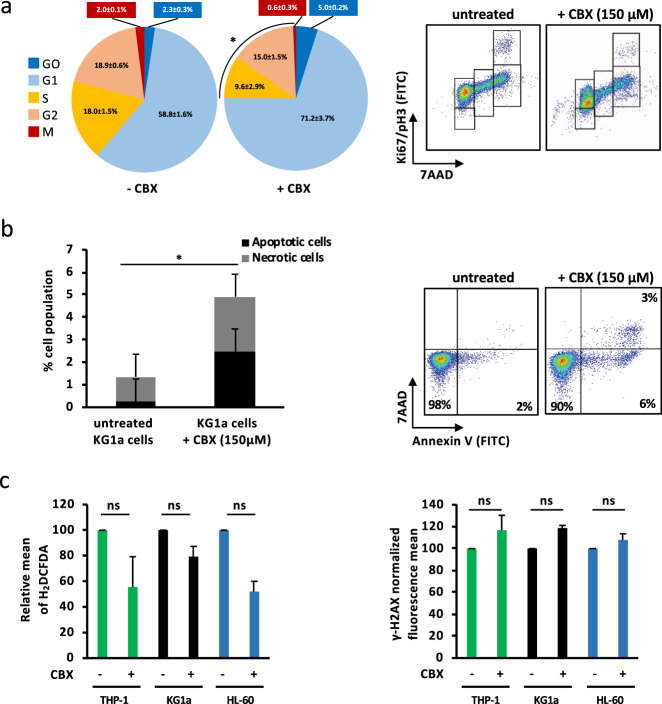
CBX affects AML cell cycle and induces apoptosis. **a** CBX (150 µM, 48 h) reduces AML cell proliferation by reducing the number of leukemic cells in S/G2/M phases (*n* = 3). **b**, **c** Treatment with CBX induced AML cells apoptosis but did not affect ROS levels in AML cells neither induced DNA damage (*n* = 3). Results are expressed as mean ± SEM. *P < 0.05; ns nonsignificant

In order to investigate whether CBX could affect the growth of AML cells, various leukemia cell lines were treated with different concentrations of CBX. MTT and trypan blue exclusion assays showed that CBX reduced AML cell growth and viability, respectively. For instance, KG1a cell growth was significantly inhibited by 50% at 150 µM of CBX after 48 h of exposure. In addition, concentrations above 200 µM of CBX caused a major decrease in KG1a cell growth at 24, 48, and especially 72 h of treatment (Fig. 2a). Moreover, 48 h exposure of six AML cell lines representing different FAB subtypes to various doses of CBX showed a 50% decrease of cell growth at 100–150 µM of CBX (Fig. 2b). Of note, MTT results were reinforced by trypan blue exclusion assays which confirmed that CBX treatment (150 µM, 48 h) significantly reduced the viable cell numbers of various AML cell lines by around 50–80% (Fig. 2c). Finally, exposure to CBX (150 µM, 48 h) of primary blast cells isolated from BM samples of five AML patients (Supplementary Table 2) significantly decreased the viable cell numbers (Fig. 2d). In summary, CBX has displayed deleterious effects on AML cells in a time- and dose-dependent manner, with an IC_50_ around 150 µM. At this concentration, the antiproliferative effects of CBX can be explained by cell cycle inhibition and induction of apoptosis. Indeed, CBX significantly reduced the percentage of cells engaged in the cell cycle (reduction of ≈36% of S, G2, and M phases) (Fig. 3a), and significantly induced apoptosis and necrosis (Fig. 3b) without concomitant increase in ROS levels nor DNA damage (Fig. 3c).

**Fig. 4:**
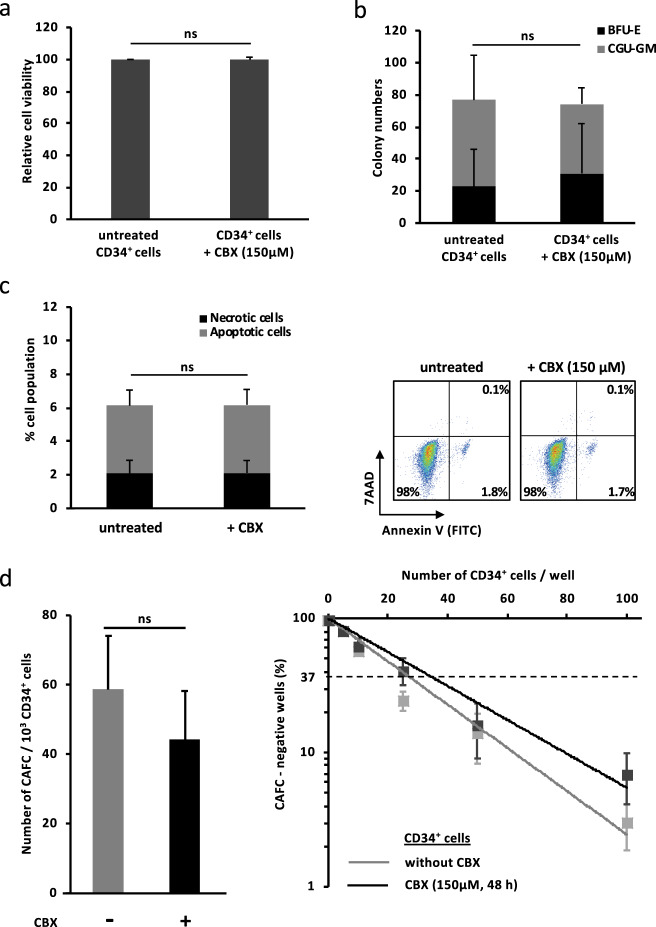
CBX has no effect on normal BM CD34^+^ cells. **a** CBX treatment (150 µM, 48 h) showed no effect on BM CD34^+^ cell numbers determined by trypan bleu exclusion assay. The results are normalized to untreated cells (*n* = 5). **b** CBX treatment did not reduce the colony-forming capacity of CD34^+^ progenitors. Results represent the number of forming colonies (*n* = 3). **c** CBX treatment (150 µM, 48 h) did not induce BM CD34^+^ cell apoptosis nor necrosis. The results were obtained using annexin V-FITC and 7AAD staining (*n* = 5). Results are reported as mean ± SEM. ns nonsignificant. **d** CBX treatment (150 µM, 48 h) did not modify the frequency of primitive progenitors (CAFC day 35), quantified by limiting dilution assay (*n* = 3). ns nonsignificant

The effects of CBX treatment on normal BM hematopoietic cells was evaluated by exposing freshly isolated normal BM-CD34^+^ cells to CBX (150 µM, 48 h). CBX did not modify cell number and viability (Fig. 4a) and did not decrease the number of BM-CD34^+^ clonogenic progenitors (Fig. 4b). Moreover, CBX did not promote cell apoptosis or necrosis of these normal progenitors (Fig. 4c). Finally, CAFC quantification was performed by limiting dilution assays on murine MS5 cell line, and CBX did not modify the frequency of CAFC in BM-CD34^+^ cells (Fig. 4d).

### CBX reduces AML cell metabolic activity

**Fig. 5:**
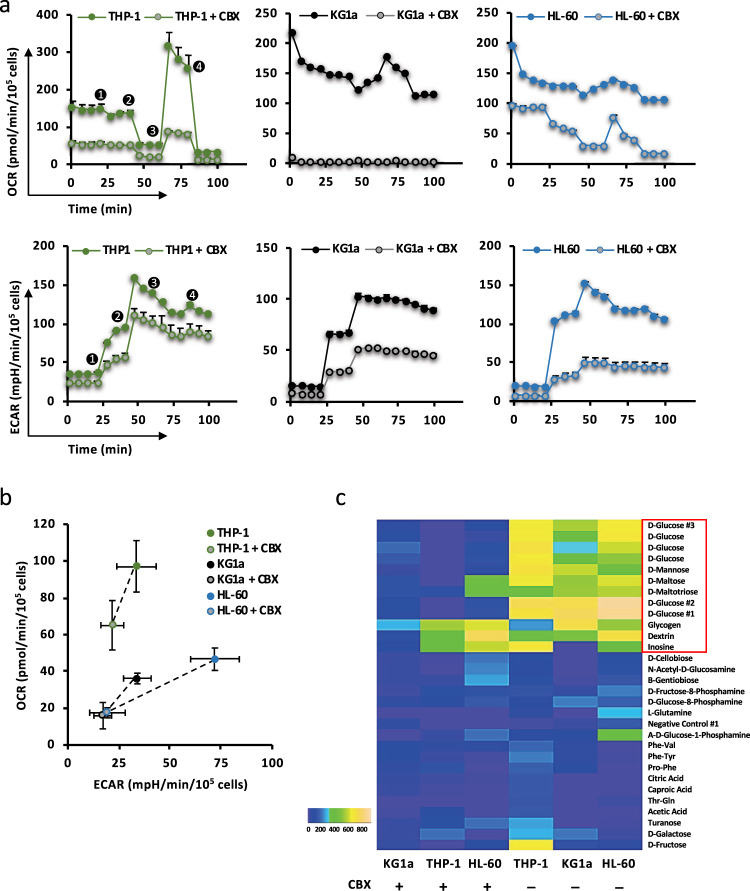
CBX reduces AML cell metabolic activity. The oxygen consumption rate (OCR) and extracellular acidification rate (ECAR) were concomitantly analyzed by the Seahorse XFe96 Bioanalyzer in three AML cell lines representative of the three groups of response to Ara-C, exposed or not to CBX (150 µM, 48 h). **a** Representative experiments. The sequential injections were: (1) glucose 10 mM, (2) oligomycin 1 μM, (3) DNP 100 μM, and (4) a mix of rotenone 0.5 μM and antimycin A 0.5 μM. **b** THP-1, KG1a, and HL-60 cells showed a concomitant decrease in their OCR and ECAR values after exposure to CBX (*n* = 3). **c** The effect of CBX on the ability to metabolize 367 substrates was measured by OmniLog® analyzer, revealing a decrease of metabolism capacity of AML cells treated with CBX. The major metabolic modifications induced by CBX concerned the metabolism of glucose, mannose, maltotriose, maltose, glycogen, and dextrin. Heatmap shows data with AUC > 150 in at least one condition. The area under the curve (AUC) of each substrate consumption was measured using the OmniLog® software and normalized in R using opm package to the control conditions, and all AUC values are presented as heatmap

To assess the variations of AML cell metabolism after 48 h exposure to CBX, three AML cell lines were chosen according to their chemoresistance: THP-1 as resistant, KG1a as intermediate, and HL-60 as sensitive cells. Following exposure to CBX, the cells’ OCR and ECAR were measured in various conditions as described above. The basal metabolism of the three cell lines was determined to be OXPHOS, intermediate, and glycolytic, respectively. CBX treatment decreased basal OXPHOS by 36%, 50%, and 62% (Fig. 5a, b), and this effect was mainly explained by the decrease in ATP-linked OCR (nonsignificant effect on proton leak) by 37%, 60%, and 62% for THP-1, KG1a, and HL-60, respectively (Supplementary Fig. S1a). Moreover, maximal respiration after CBX exposure was highly decreased, revealing a major perturbation in mitochondrial functions (Supplementary Fig. S1b). Considering glycolysis, CBX induced a decrease in ECAR measurements (Fig. 5a, b). This lowered level of glycolysis was not compensated by an increase in the glycolytic reserve, unused in the basal state but that could be recruited in response to increases in ATP demand, inducing a decreased glycolytic capacity (Supplementary Fig. S1c).

THP-1, KG1a, and HL-60 cell lines showed, respectively, 35%, 56%, and 73% reduced glycolysis levels after exposure to CBX (Fig. 5b). Moreover, the energy metabolism of these cells in the minutes following CBX injection revealed a decrease in their mitochondrial respiration with a concomitant but transient increase in glycolysis (Supplementary Fig. S1d). Thus, the energy metabolism of leukemic cells was globally altered by CBX treatment. Moreover, CBX did not affect the OXPHOS neither the glycolytic capacities of normal BM-CD34^+^ cells (Supplementary Fig. S2).

To investigate more precisely CBX effect on cell metabolism, screening of a large range of metabolized nutrients was performed with the OmniLog® analyzer as described above (Supplementary Fig. S3). After removal of unmetabolized substrates, CBX induced a reduction of D-glucose, D-mannose, D-maltose, maltotriose, glycogen, and dextrin metabolism, without compensation by overuse of other substrates (Fig. 5c). The CBX-induced reduction of leukemic cells metabolism identified by the decrease in OCR and ECAR is consistent with these Omnilog® results.

### Carbenoxolone and cytarabine have synergistic antileukemic effect on human AML cells

Since CBX showed antileukemic activity, we investigated the combination effect of CBX and the antileukemic agent Ara-C by isobologram analyses. To this end, individual IC_50_ doses for CBX and Ara-C were determined and then plotted on the *x* and *y* axes, according to method previously described [38]. The isobolograms of AML cell lines showed a synergistic effect between the two drugs (Supplementary Fig. S4). Moreover, three different response profiles to Ara-C were obtained, corresponding to the chemosensitivity of cell lines, THP-1 and MV4-11 being resistant, KG1a and KG-1 intermediate, and HL-60 and Molm-13 sensitive. In all cases, a synergistic effect of CBX and Ara-C was observed, independently from the resistance level to Ara-C of AML cells.

### CBX has no effect on the viability and differentiation of BM-MSCs

The chemosensitivity of leukemic cells is known to be modulated by the contact with the BM niche, where they interact with MSCs notably through gap junctions. Before performing coculture experiments, we tested CBX impact on normal primary BM-MSCs. The cells were exposed to various doses of CBX for 48 h. Doses up to 150 µM CBX did not affect the viability of the cells (Supplementary Fig. S5a), in which apoptosis and necrosis where unchanged compared with control conditions (Supplementary Fig. S5b), while higher doses of CBX (>200 µM) decreased viability by promoting apoptosis. Moreover, CBX did not affect the differentiation capacities of BM-MSCs into adipocytes, chondrocytes, or osteoblasts (Supplementary Fig. S5c). Finally, CBX treatment had no toxic effect on leukemic BM-MSCs since it did not induce apoptosis in primary BM-MSCs isolated from AML patients (Supplementary Fig. S5d).

### CBX reduces the protective effect of the stroma on AML cells

**Fig. 6:**
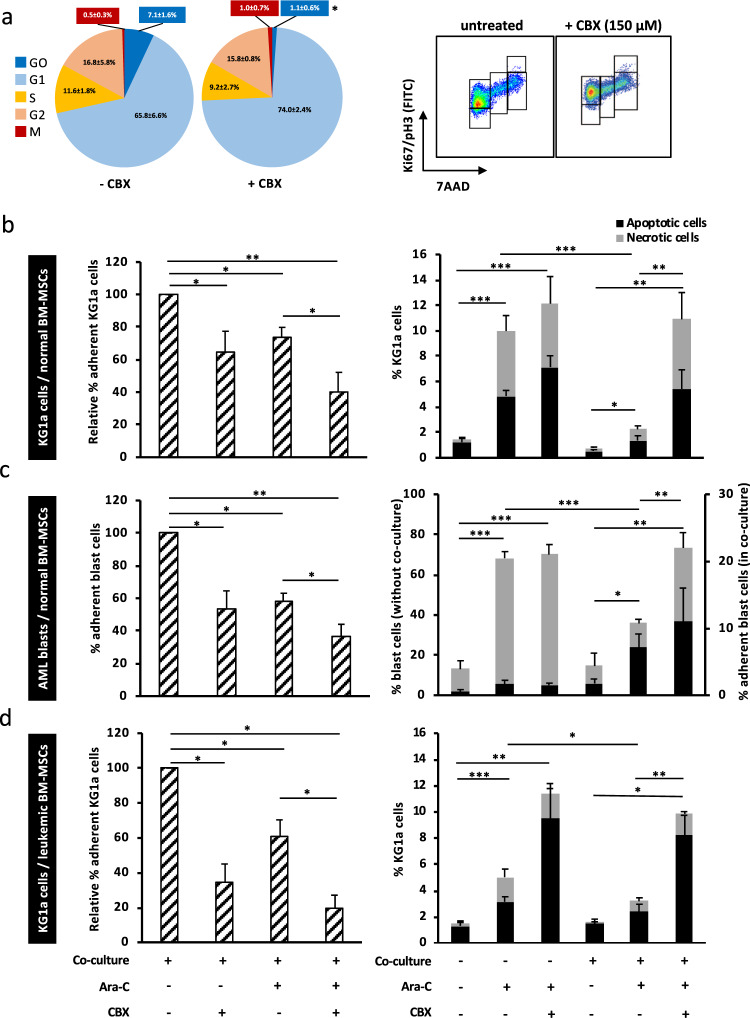
CBX reduces the BM-MSC-induced chemoresistance of AML cells to cytarabine. Cocultures experiments of leukemic cells and BM-MSCs were performed for 48 h with CBX (150 µM) and/or Ara-C (1 µM). **a** CBX decreased the percentage of quiescent leukemic cells (G0 phase) in contact with BM-MSCs and did not reduce the percentage of cells actively engaged in the cell cycle (S, G2, and M phases), conversely to its effect on isolated leukemic cells (*n* = 5). **b** (Left) Ara-C and CBX reduced the adhesion of KG1a cells to normal BM-MSCs. The percentage of adherent KG1a cells (CD45^+^) was reduced after adding Ara-C and/or CBX to the coculture system (*n* = 5). **b** (Right) Normal BM-MSCs protected KG1a cells against the proapoptotic effect of Ara-C, and CBX treatment reduced BM-MSC-antiapoptotic protection. Apoptosis and necrosis were studied in CD45^+^ CD90^−^ adherent KG1a cells (*n* = 5). **c** (Left) Ara-C and CBX reduced the adhesion of primary AML blast cells on normal BM-MSCs. The percentage of adherent leukemic cells (CD45^+^) was reduced after adding Ara-C and/or CBX to the coculture system (*n* = 5). **c** (Right) Normal BM-MSCs protected primary AML blast cells against the proapoptotic effect of Ara-C, and CBX treatment reduced BM-MSC-antiapoptotic protection. Apoptosis and necrosis were studied in CD45^+^CD90^−^ adherent AML blast cells (*n* = 5). **d** (Left) Ara-C and CBX reduced the adhesion of KG1a cells on leukemic BM-MSCs. The percentage of adherent leukemic cells (CD45^+^) was reduced after adding Ara-C and/or CBX to the coculture system (*n* = 5). **d** (Right) Leukemic BM-MSCs protected KG1a cells against the proapoptotic effect of Ara-C, and CBX treatment reduced MSC-antiapoptotic protection. Results are expressed as mean ± SEM. *P < 0.05; **P < 0.01; ***P < 0.001

Coculture experiments were performed with KG1a or primary AML blast cells, together with normal or AML BM-MSCs, to evaluate the impact of CBX exposure on niche-induced chemoresistance to Ara-C. CBX induced a sixfold decrease in the percentage of quiescent leukemic cells (G0 phase) in contact with normal BM-MSCs, an observation consistent with a direct effect on gap junctions assembly (Fig. 6a). Moreover, in this context, CBX did not reduce the percentage of leukemic cells actively engaged in the cell cycle (S, G2, and M phases), at variance to its effect previously shown on isolated leukemic cells (reduction of ≈36% of S, G2, and M phases). The adhesion of KG1a cells to normal BM-MSCs was decreased after Ara-C treatment (−27 ± 6%). This decrease was amplified after CBX exposure (−35 ± 11%), and even more by concomitant Ara-C and CBX treatment (−60 ± 12%) (Fig. 6b left). Similar results were obtained using primary AML blast cells (−42 ± 5%, −47 ± 10%, and −64 ± 7%, respectively) (Fig. 6c left) and KG1a cocultured with AML BM-MSCs (−65.5 ± 10%, −40 ± 9%, and −80 ± 7%, respectively) (Fig. 6d left).

As expected, without BM-MSCs, Ara-C induced apoptosis/necrosis of KG1a and primary AML blast cells (11 ± 1% and 68 ± 2%, respectively). This effect was ≈sixfold reduced by contact with normal BM-MSCs (2 ± 1% and 11 ± 2%, respectively), confirming the acquired niche-induced chemoresistance. Interestingly, cotreatment with CBX counteracted this phenomenon, by enhancing Ara-C-induced apoptosis/necrosis of KG1a and primary AML blast cells in contact with BM-MSCs (11 ± 3% and 22 ± 6%, respectively) (Fig. 6b right, 6c right). Similar results were obtained when KG1a cell lines were cocultured with primary AML BM-MSCs (9.8 ± 1%) (Fig. 6d right).

In summary, combination treatment of Ara-C with CBX increased the antileukemic effect of Ara-C especially by reducing the chemoresistance triggered by BM-MSCs. CBX effect was promoted by direct contact of AML cells with BM-MSCs, revealing a potential role of gap junctions in the regulation of chemoresistance.

### CBX reduces heterocellular communication between KG1a and BM-MSCs

To investigate the role of CBX as a disruptor of gap junctions between leukemic cells and MSCs, calcein transfer assay was performed. BM-MSCs were labeled with calcein-AM and cocultured with unlabeled KG1a. Transfer of calcein from BM-MSCs to KG1a was detected in control conditions, confirming the assembly of functional gap junctions. Exposure to CBX decreased calcein transfer by more than 50% (Supplementary Fig. S6).

To assess the variations of AML cell metabolism in contact with BM-MSCs, KG1a cells were FACS-sorted after 48 h of coculture with stromal cells. CBX induced a decrease in OCR and ECAR in adherent KG1a cells (Supplementary Fig. S7a–c). The effect on ECAR was higher and a major decrease in glycolytic ATP production was observed (decrease of 62 and 43% in glycolytic and mitochondrial ATP production, respectively). Therefore, the inhibition of energy metabolism induced by CBX was also observed in AML cells directly interacting with BM-MSCs. This lower capacity to produce energy explains the higher sensitivity of leukemic cells to chemotherapy. The CBX-induced disruption of functional gap junctions in the leukemic niche probably participates to this process by limiting the transfer and metabolic support offered by the microenvironment. This supports that regulation of niche-induced chemoresistance of AML cells involves gap junctions which could be targeted by CBX, in accordance with all previous data.

The antileukemic effects of CBX cannot be explained by off target effects on 11β-hydroxysteroid deshydrogenase (Supplementary Fig. S8a) [39]. HSD11B1 mRNA was not detectable in 22 AML cell lines and 11β-hydroxysteroid deshydrogenase protein was not found or induced by CBX in six AML cell lines (Supplementary Fig. S8b). Finally, cortisone and cortisol quantification in the supernatant of AML cells did not reveal any difference after CBX exposure (Supplementary Fig. S8c).

### Mice experiments

**Fig. 7:**
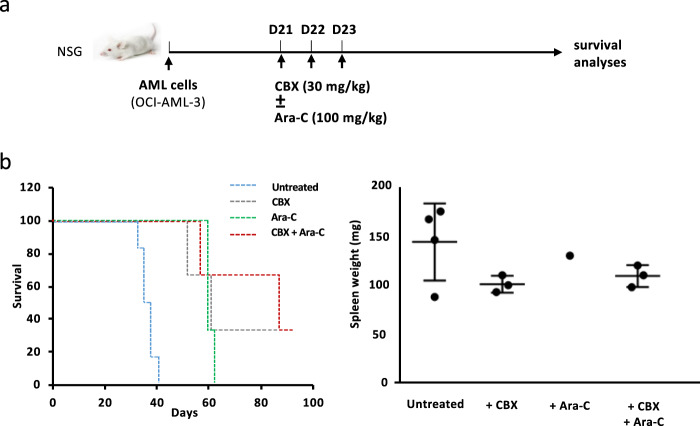
CBX increases the survival of treated mice in a xenogenic human AML model. **a** Experimental procedure of in vivo experiments with OCI-AML-3 human AML model (at least three for each condition). **b** (Left) CBX treatment improved the mice survival. **b** (Right) AML induced major splenomegaly in the untreated group, which was not observed in treated mice

To establish the potential therapeutic utility of CBX in combination with Ara-C, we investigated their effect in vivo using the OCI-AML-3 human AML model in NSG mice (Fig. 7a). Survival analyses served as an indicator of CBX effect on the AML aggressiveness. Untreated mice died after 32–41 days. Treatments with Ara-C alone, CBX alone, and even more CBX and Ara-C infusions significantly improved mice survival as shown by Kaplan–Meir analyses (Fig. 7b left). In line with these results, CBX and Ara-C infusions induced a threefold decrease in BM blastosis in untreated mice (50 ± 7% of human CD45^+^ cells), in which a major splenomegaly was observed in contrast to treated mice (Fig. 7b right). Finally, AML cells also induced an increase in liver size. CBX treatment limited the hepatomegaly, the best results being observed when it was combined with Ara-C. Altogether, these results, obtained with an aggressive human AML model, support the in vivo antileukemic activity of CBX.

## Discussion

In this study, we established a large-scale expression profile of the Cx family in leukemic cells compared with normal CD34^+^ hematopoietic progenitors and in leukemic BM-MSCs compared with normal BM-MSCs. Interestingly, the same Cxs were highly expressed in leukemic and normal hematopoietic cells (notably Cx25, Cx31.9, and Cx59), whereas leukemic BM-MSCs differed from their normal counterpart by specifically overexpressing numerous Cxs, Cx25, and seven others. These new interesting data emphasize the possibility of specific gap junction interactions between AML cells and BM-MSCs in the leukemic niche, probably involving homotypic Cx25-gap junctions. In line with this, Cx25 has been described as potentially implicated in cell–cell communication between leukemic cells [40].

**Fig. 8:**
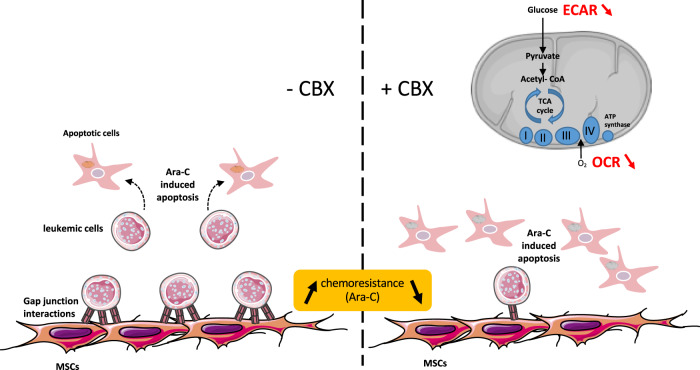
Schematic model of the antileukemic effect of CBX, a disruptor of gap junctions, in the context of the leukemic niche. CBX decreases the proliferation of leukemic cells by promoting apoptosis via global extinction of energy metabolism (oxidative phosphorylation and glycolysis). The most important antileukemic effect is observed at the level of the leukemic niche, since CBX reduces the chemoresistance to Ara-C triggered by BM-MSCs

Furthermore, we disclosed a key role of gap junctions in the chemoresistance of AML leukemic cells, counteracted by using a disruptor of gap junction assembly (Fig. 8). CBX displayed an antileukemic activity on AML cells, amplified on leukemic cells resisting to Ara-C-induced apoptosis when residing in the niche modelized by coculture with normal or leukemic BM-MSCs. CBX was shown to induce an extinction of the energy metabolism of leukemic cells. Its proapoptotic effect synergized with antimitotic Ara-C to promote cell death. Interestingly, CBX had no deleterious effects on normal BM CD34^+^ hematopoietic progenitors. The in vitro antileukemic activity of CBX was reinforced by in vivo experiments showing that CBX improves survival and limits the leukemic infiltration of the liver and spleen of mice developing an aggressive human AML.

The BM microenvironment plays a major role in tumorigenesis and supports leukemic cells anchoring [41] by providing nutrients and survival signals to tumor cells [42] while favoring their maintenance in a quiescent stage thereby limiting chemosensitivity. Little is known regarding the role of Cxs in the AML niche, and almost exclusively concerns Cx43. In t(8;21) AML, and thus probably through with the AML1-ETO fusion protein, an increase in Cx43 expression has been reported via c-Jun disruption [43, 44]. The expression level of Cx43 in AML BM-MSCs postchemotherapy was significantly higher and similar to normal levels than in primary AML BM-MSCs in another study [45]. Later, coculture of Jurkat cells with Cx43-transfected stromal cells was shown to increase leukemic cell proliferation, apoptosis, and chemosensitivity [46].

A tumor suppressor role of gap junction channels and Cxs has been reported, and the upregulation of Cxs in solid cancer cells restores cell growth and differentiation [24, 47]. To date, little is known about the role of gap junctions and Cxs in a probable direct interaction of AML cells with their microenvironment. In this study, performed with human AML cells and primary BM-MSCs, we established the implication of gap junctions in the regulation of leukemic cell growth and chemoresistance. CBX is known as a global disruptor of gap junction assembly, used to study the nonspecific blockage of Cxs, and our results revealed an antileukemic effect which was synergistic with Ara-C. CBX blocks the assembly of Cxs in the gap junctions and regulates the transfer of mi-RNA and several metabolites such as Ca^2+^ and glucose. Moreover, it has been described that CBX inhibits 11β-hydroxysteroid deshydrogenase [39], but this enzyme was not expressed in the leukemic cells. Cancer cell survival is linked to the cell metabolism pathways including glycolysis, OXPHOS as well as amino acids and fatty acids metabolism [48]. Interestingly, the antileukemic effect of CBX in our experiments was characterized by the extinction of energy metabolism of AML cells regardless of their level of chemoresistance to Ara-C. CBX reduced oxygen phosphorylation, ATP production, and maximal respiration of AML cells. Glycolysis was also altered, and the metabolism of such carbohydrates as mannose, glucose, and maltose was decreased. This is of major importance since glucose metabolism, transporters, and glycolytic enzymes are necessary to support cancer cell proliferation [49, 50].The proapoptotic effects of CBX on leukemic cells could be explained by the relationship between cell metabolism and induction of apoptosis. The mitochondrion is considered as a platform to regulate cell fate, and extinction of energy metabolisms having become inadequate for cellular demands induces apoptosis [51].

Accumulating evidence shows the critical role of the BM niche in the maintenance and retention of leukemic cells. The involvement of direct cell–cell interactions appears to be major in the regulation of leukemic initiation, progression, and response to therapy. Direct interaction with BM-MSCs has been reported to decrease AML cell apoptosis by regulating mTOR and PI3K/Akt pathways [52] and upregulating the antiapoptotic effect of Bcl2 [19], thereby providing cell protection from chemotherapy agents [53]. Paraguassu-Braga et al. reported the effects of gap junction disruption between murine lymphoblastic and stromal cell lines (CCRF-CEM and S17 cells, respectively) [54]. In this study, the quiescence of CCRF-CEM cells and their resistance to methotrexate was promoted by contact with S17 cells and the use of CBX reduced these effects. Our study performed using a human primary AML model of cocultures between leukoblasts and primary normal or leukemic BM-MSCs allowed us to show that disruption of gap junctions by CBX reduced the protective effect of the human BM-niche, by reducing the chemoresistance of AML cells promoted by BM-MSCs. Interestingly, our data showed that CBX does not present any cytotoxic effect on normal BM-CD34^+^ hematopoietic progenitors.

The involvement of gap junction disruption in our models is reinforced by two major arguments: (i) CBX inhibited the transfer of calcein between BM-MSCs and AML cells and (ii) its effects on coculture experiments was cell-contact dependent since it reduced the G0 phase only in AML cells in contact with BM-MSCs.

In conclusion, this report identified a new molecular Cx signature that could be involved in the formation of specific functional gap junctions playing a role in leukemia pathophysiology. We describe the involvement of gap junctions in the acquired chemoresistance triggered by the leukemic niche. Moreover, we demonstrate that these interactions are targetable by the pharmacological inhibitor CBX, a drug already evaluated in the treatment of gastric/duodenal ulcer [55, 56]. This gap junction disruptor could be of particular clinical interest in AML treatment since it was also shown here that it synergizes with cytarabine in AML cells, yet without deleterious effects on normal BM-hematopoietic progenitors.

## Materials and methods

### Cells and reagents

Human AML cell lines were purchased from the European Collection of Authenticated Cell Cultures (ECACC, Wiltshire, UK) and cultured as previously described [38]. The FILOtheque AML (# BB-0033-00073, Cochin hospital, Paris, France), tumor bank of the FILO (French Innovative Leukemia Organization) group, provided 39 annotated samples. All patients had provided their informed consent for cell banking according to the Declaration of Helsinki, and the study was approved by the French Ministry of Higher Education and Research (authorization number # DC-2008-308). Leukemic cells were cultured in a culture medium described for ex vivo maintenance [57] and AML BM-MSCs were derived from 19 AMLs samples. Primary normal BM-MSCs and BM-CD34^+^ were isolated from healthy donors (without any hematological disorder) undergoing orthopedic surgery (University Hospital, Tours, France) after informed consent and following a procedure approved by the local ethical committee. BM-MSCs were amplified as previously described [58]. CD34^+^ cells were enriched using a magnetic bead separation kit (MiniMACS®, Gladbach, Germany), as previously described [21] or using BD FACSMelody™ cell sorter (BD Biosciences, San Jose, CA, USA). The overall purity of selected CD34^+^ cells was >95% and the cells were then cultured in vitro as previously described [59].

### Coculture experiments

The culture medium of BM-MSCs was renewed without FGF-2 on the day before leukemia cells/BM-MSCs coculture. At day 0, leukemia cells were seeded at 1.5 × 10^4^ cells/cm^2^ alone or over a BM-MSC layer, and the experiments were performed for 2 days at 37 °C, 5% CO_2_ with or without treatments. CBX disodium salt (Merck KGaA, Darmstadt, Germany) was freshly diluted in culture medium and used at different concentrations (0–300 μM). Ara-C (Fresenius Kabi Oncology Plc, Hampshire, UK) was diluted in culture medium and used at 1 μM. Exposure to CBX and/or Ara-C was performed for up to 72 h.

### RNA extraction and quantitative reverse transcription PCR (RT-qPCR)

Total cellular RNA was extracted with a Maxwell RNA purification kit (Promega, Madison, WI, USA), and quantified using a NanoDrop^TM^ Lite spectrophotometer (ThermoFisher Scientific, Waltham, MA, USA). RNA purity was analyzed using an Agilent 2100 Bioanalyzer (Agilent Technologies, Santa Clara, CA, USA). Five micrograms of total RNA from each sample were reverse transcribed using the SuperScript® VILO^TM^ cDNA Synthesis kit (Invitrogen, Carlsbad, CA, USA). Primers designed according the Roche Universal Probe Library (UPL) were validated with Stratagene cDNA mix (Agilent Technologies) (Supplementary Table 1). RT-qPCR reactions were performed using a LightCycler® 480 Probes Master (Roche Life Science, Basel, Switzerland). Samples were subjected to an initial denaturation step (5 min, 95 °C), followed by 45 PCR cycles (10 s, 95 °C, then 30 s, 60 °C) and a final cooling step (40 °C, 30 s). All reactions were run concomitantly in triplicates, and results analyzed using the Cycle threshold (Ct) values. The geometric Ct mean of human *ACTB*, *YWHAZ*, *RPL13A, EF1A*, and *GAPDH* genes were used as endogenous control to normalize the expression of target genes: ΔCt = “Ct target” − “Ct reference”.

### Apoptosis/necrosis assays

Cells were harvested at day 2 of coculture and apoptosis was studied by flow cytometry using a FACS CantoII cytometer (BD Biosciences). Primary BM-MSCs and AML cells were discriminated by surface expression of CD90 (APC, BD Biosciences) and CD45 (violet, BD Biosciences), respectively. Apoptosis/necrosis was quantified after staining with annexin V and 7AAD (Annexin V FITC/7-AAD kit, IM3614, Beckman Coulter, Brea, CA, USA), as we previously described [60].

### Flow cytometry analysis of cell cycle

Detailed cell cycle analysis of KG1a cells in each condition was performed by quantifying G0, G1, S, G2, and M phases according to a method of nucleic acids labeling previously published by our group [61]. Cells of interest were stained as above with APC-conjugated anti-CD90 and violet-conjugated anti-CD45 antibodies (BD Biosciences). Analyses were performed on a FACS Canto II cytometer.

### MTT assay

MTT (Merck KGaA) assays and isobolograms were performed as we previously described [38].

### Metabolic analyses

Energy metabolism was quantified using a Seahorse XFe96 Analyzer (Agilent Technologies). After 48 h of CBX treatment, 10^5^ viable cells were plated in each well. Oxygen consumption rate (OCR) and extracellular acidification rate (ECAR) measurements were carried out in a substrate-free base medium (XF Base Medium) supplemented with 2 mM glutamine (Gibco, Carlsba, CA, USA). OCR and ECAR values are presented as pmol/min/10^5^ cells and mpH/min/10^5^ cells, respectively. Sequential injections of glucose (10 mM), oligomycin (1 μM), dinitrophenol (DNP), or rotenone/antimycin A (0.5 μM) from Merck KGaA were used to determine the major metabolic parameters. A similar strategy was used to assess the immediate effect of CBX on AML cells by direct injection during Seahorse analyses, CBX being injected instead of glucose, which was added in the base medium (10 mM).

Metabolic profiling was studied by using the Omnilog® Phenotype Microarray^TM^ system (Biolog, Hayward, CA, USA) evaluating the cell’s ability to metabolize 367 substrates [62]. Leukemic cells were cultured for 24 h in PM-M1, PM-M2, PM-M3, and PM-M4 plates (Biolog) in a substrate-free base medium (IFM1 medium, Agilent) supplemented with 0.3 mM glutamine, 5% FCS, 100 U/mL penicillin, and 100 μg/mL streptomycin. CBX (150 μM) was added in the last 6 h before measurements, and the MB dye was then added according to the manufacturer’s instructions. Measurements were then performed during 24 h with the Omnilog® automated incubator-reader.

### Statistical analyses

Statistical analyses were performed using nonparametric tests (Mann–Whitney or Kruskall–Wallis test followed by Dunn post hoc test for multiple comparison). Survival curves were compared using log-rank test. Statistics were computed with R software (version 3.5.0, https://www.r-project.org/). Sample size and *P* values are indicated in the legend of each figure.

## Supplementary information

Supplemental material_Text

Supplemental material_Figures

Supplemental material_Tables
