# Correction: Disruption of gap junctions attenuates acute myeloid leukemia chemoresistance induced by bone marrow mesenchymal stromal cells

**DOI:** 10.1038/s41388-019-1089-7

**Published:** 2019-11-07

**Authors:** Farah Kouzi, Kazem Zibara, Jerome Bourgeais, Frederic Picou, Nathalie Gallay, Julie Brossaud, Hassan Dakik, Benjamin Roux, Sophie Hamard, Louis-Romee Le Nail, Rita Hleihel, Amelie Foucault, Noemie Ravalet, Florence Rouleux-Bonnin, Fabrice Gouilleux, Frederic Mazurier, Marie C. Bene, Haidar Akl, Emmanuel Gyan, Jorge Domenech, Marwan El-Sabban, Olivier Herault

**Affiliations:** 1 CNRS ERL7001 LNOx “Leukemic Niche & Redox Metabolism”, Tours, France; 2 EA7501 GICC, University of Tours, Faculty of Medicine, Tours, France; 3 PRASE, DSST, Lebanese University, Beirut, Lebanon; 4 Biology Department, Faculty of Sciences, Lebanese University, Beirut, Lebanon; 5 Department of Biological Hematology, Tours University Hospital, Tours, France; 6 Department of Nuclear Medicine, Bordeaux University Hospital, Pessac, France; 7 Department of Surgical Orthopedia, Tours University Hospital, Tours, France; 8 Department of Anatomy, Cell Biology, and Physiological Sciences, Faculty of Medicine, American University of Beirut, Beirut, Lebanon; 9 Department of Internal Medicine, Faculty of Medicine, American University of Beirut, Beirut, Lebanon; 10 Department of Biological Hematology, Nantes University Hospital, CRCINA, Nantes, France; 11 Department of Hematology and Cell Therapy, Tours University Hospital, Tours, France

**Keywords:** Cancer microenvironment, Acute myeloid leukaemia

**Correction to: Oncogene**

10.1038/s41388-019-1069-y

The original version of this article omitted the following from the Acknowledgements:

This research was also supported by grants to KZ (UL and L-CNRS).

This has now been corrected in both the PDF and HTML versions of the Article.

