# Supplementary material for: Disruption of gap junctions attenuates acute myeloid leukemia chemoresistance induced by bone marrow mesenchymal stromal cells
Source: Oncogene. 2019 Oct 24;39(6):1198–212. doi: 10.1038/s41388-019-1069-y (PMC7002301; doi:10.1038/s41388-019-1069-y)
Supplement: Supplementary file 1 — Supplemental material_Text [file 41388_2019_1069_MOESM1_ESM.docx]

**Supplementary Information**

**Disruption of gap junctions attenuates acute myeloid leukemia chemoresistance induced by bone marrow mesenchymal stromal cells**

**Supplementary Materials and Methods**

**ROS and DNA damage assessment**

After exposure to CBX, ROS measurements were performed as previously described [s1]. To assess cell DNA damage, cells were fixed and permeabilized as described [s2] and stained with 1 µL of anti- γH2AX (Millipore, Burlington, MA, USA) for 3 h on ice. Analyses were performed with BD Accuri™ C6 flow cytometer (BD Biosciences).

**Cobblestone area-forming cell assay**

Cultures were initiated on preestablished stromal layers from MS-5 cells in 96-well plates for cobblestone area-forming cell (CAFC) quantification at limiting dilution. Cells were incubated for 2 days as previously described [41] (± CBX 150 μM) and then plated into each of the 96 wells at 6 different dilutions (range 1–100 CD34^+^ cells/well) in 0.2 mL of Myelocult^TM^ H5100 medium (StemCell Technologies, Vancouver, Canada). After 5 weeks of incubation with weekly half medium changes, the percentage of wells with at least one phase-dark cobblestone area (at least twenty cells seeding within the stromal layer) was determined using an inverted microscope. The percentage of negative wells (no cobblestone area detectable) was determined and the frequency of CAFC in the starting CD34^+^ cell population calculated by Poisson statistics as the reciprocal of the cell concentration that gave 37% negative wells.

**Protein extraction and immunoblots**

Cells were lysed in 10 mmol/L HEPES (pH 7.9), 10 mmol/L KCl, 0.1 mmol/L EDTA, 0.1 mmol/L EGTA, and 10% [vol/vol] NP40 containing protease inhibitors. Western blot analyses were done as described [s1] using polyclonal antibodies against 11β-hydroxysteroid dehydrogenase type 1 (ab39364, Abcam, Cambridge, UK) and corresponding recombinant peptide (ab114301, Abcam) was used as positive control.

**Quantification of cortisol and cortisone levels by mass spectrometry**

After collection of supernatant of AML cells exposed to not to CBX (150 µM, 48 h), 50 μL of an internal standard solution (deuterated cortisol 500 ng/mL, Alsachim, Illkirch-Graffenstaden, France) was added to 10 mL of supernatant, followed by extraction with 20 mL dichloromethane. Organic phase was evaporated in a heated water bath (40°C) under nitrogen flow, re-dissolved in 100 μL of a 50:50 methanol-water solution, and then subjected to LC/MS analysis (5500 QTRAP, Sciex, Framingham, MA, USA) to determine cortisol and cortisone relative concentrations.

**Xenograft animal studies**

This study was approved by the Institutional Animal Care and Utilization Committee of the American University of Beirut. Mice were obtained from Jackson Laboratory (Bar Harbor, ME, USA) and housed under pathogen-free conditions with constant temperature and humidity control. Six to eight weeks old mice were used. OCI-AML-3 cells were grown in minimum essential medium–α (MEMα) supplemented with 20% fetal bovine serum and antibiotics. 2x10^6^ AML cells were injected into the tail vein of 8-week-old immune-deficient NSG mice (NOD.Cg-Prkdcscid Il2rgtm1Wjl/SzJ). On day 21 after the AML injection, mice received an intraperitoneal injection of CBX (30 mg/kg) or its combination with Ara-C (100 mg/kg) for three consecutive days. Survival was monitored on a daily basis unless animal were sacrificed on a chosen day (# on the Kaplan–Meier survival curve). Mice were euthanized by cervical dislocation after exposure to furane. Survival curves of xenografted mice (at least 3 per condition) were plotted using the Kaplan-Meier method on Graphpad Prism 5^®^ (Graphpad Software, San Diego, CA, USA). Spleens were weighed. Livers were removed and macroscopically evaluated.

**Isobologram analysis**

Isobolograms were obtained with 2×10^5^ cells/mL exposed to serial drug dilutions (CBX, Ara-C, and CBX ± Ara-C) for 48 h and absorbance (MTT reaction) was measured at 540 nm using a CLARIOstar^®^ Monochromator Microplate Reader (BMG Labtech, Offenburg, Germany).

**Dye-transfer assay**

The presence of functional gap junctions was evaluated by flow cytometry using a fluorescent dye-transfer assay with calcein-AM (Invitrogen), as previously described [s3]. BM-MSCs cells were labeled with 2 µM of calcein-AM at 37°C for 1 h in culture medium, then centrifuged and washed in serum-free medium for 30 min at 37°C in order to remove free dye and to allow for any non-de-esterified dye to leave the cells. Labeled cells were cultured with unlabeled KG1a for 3 h with or without CBX (150 µM). The leukemic cells were then harvested and labelled with anti-CD45 APC antibody (BD Biosciences) before flow cytometry analysis. The fluorescence emitted by calcein was quantified in CD45^+^ cells in the FL1 channel and data were analyzed using FlowJo^®^ software.

**Differentiation capacity of primary BM-MSCs**

Assays for osteogenic, adipogenic and chondrogenic differentiation of BM-MSCs were performed as previously described [s4].

**Supplementary Figure Legends**

**Supplementary Figure S1.**CBX reduces AML cell metabolism. Metabolic analyses were performed using Seahorse XFe96 and OmniLog^®^ analyzers. During the analysis, the cells were exposed to CBX or not, followed by sequential injections of: (1) oligomycin 1 μM, (2) DNP 100 μM, and (3) a mix of rotenone 0.5 μM and antimycin A 0.5 μM. **a** Treatment with CBX (150 µM, 48 h) reduced the basal metabolic capacities as shown by ATP production and proton leak in AML cells (*n*= 3). **b** Maximal respiration was also reduced after exposure to CBX (*n*= 3). **c** The effects of CBX on glycolysis of leukemic cell lines are summarized by a decreased glycolytic capacity (*n*= 3). **d** Representative experiment of the energy metabolism of AML cells in the minutes following CBX injection, which revealed a decrease in their mitochondrial respiration with a concomitant but transitory increase in the glycolysis. Results are expressed as mean ± SEM. * and ** indicate *P* < 0.05 and *P* < 0.01, respectively.

**Supplementary Figure S2.** CBX does not modify the metabolic activity of CD34^+^ cells. The oxygen consumption rate (OCR) and extracellular acidification rate (ECAR) were concomitantly analyzed by the Seahorse XFe96 Bioanalyzer in independent fresh samples of FACS-sorted BM CD34^+^ cells (n=2), exposed or not to CBX (150 µM, 48 h). The results were compared to AML cell lines and normalized to untreated cells (n=3). **a** Respiratory parameters: basal respiration, maximal respiration, proton leak and mitochondrial ATP production. **b** Glycolytic parameters: basal glycolysis, glycolytic capacity and glycolytic reserve. Results are expressed as mean ± SEM * indicates *P* < 0.05.

**Supplementary Figure S3.** Omnilog^®^ global results. CBX deleterious effect on AML cell metabolism was detected by analyzing 367 metabolic substrates in leukemic cells. The heatmap shows data with AUC > 150 in at least one conditions.

**Supplementary Figure S4.** Isobologram analyses reveal a synergistic effect of CBX and Ara-C on AML cells. Individual IC50 values for various doses of CBX and Ara-C (48 h), as determined by MTT assay, were calculated and plotted. The data points on the isobologram represent the actual doses of combined CBX and Ara-C treatment that result in 50% growth inhibition. When data points are positioned well below the linear curve, a synergistic anti-proliferative effect is indicated. The predicted synergism of the drugs was consistent for different AML cell lines, and 3 groups of AML cell response to Ara-C were discriminated. Each curve represents the average of independent experiments (*n =*7).

**Supplementary Figure S5.** CBX (150 µM, 48 h) does not affect the viability, apoptosis nor differentiation capacities of BM-MSCs. **a** Confluent normal BM-MSCs were treated with several concentrations of CBX for 48 h. Trypan blue exclusion assay showed a negligible decrease in BM-MSCs numbers when treated with concentrations less than 200 µM of CBX. The number of viable cells was expressed as a percentage of the control (*n*= 3). **b** CBX showed no induction of BM-MSCs apoptosis at concentrations less than 250 µM. Apoptosis and necrosis of BM-MSCs was studied by flow cytometry using Annexin V-FITC and 7AAD staining (*n*= 3). A representative experiment is presented in the lower panel. **c** Treatment with CBX did not affect the differentiation capacities of BM-MSCs into adipocytes (Adipo), osteoblasts (Osteo) or chondrocytes (Chondro). A representative experiment is shown. Scale bars correspond to 50 µm. **d** CBX did not induce apoptosis in leukemic BM-MSCs (*n*= 3). Results are expressed as mean ± SEM. ns, non-significant; * indicates *P* < 0.05.

**Supplementary Figure S6.** CBX reduces hetero-cellular communications between KG1a and BM-MSCs. **a** Schematic illustration of the experimental strategy. **b** CBX limited calcein transfer from calcein-labelled BM-MSCs to unlabeled KG1a in co-culture system. **c** Representative experiment of calcein transfer inhibition by CBX. Quantification of cell communication was performed by measuring the mean fluorescent intensity (MFI) of calcein. Results are expressed as mean ± SEM (*n* = 3). * indicates *P* < 0.05.

**Supplementary Figure S7.** CBX reduces the metabolic activity of KG1a cells in contact with primary BM-MSCs. The oxygen consumption rate (OCR) and extracellular acidification rate (ECAR) were concomitantly analyzed by the Seahorse XFe96 Bioanalyzer in FACS-sorted KG1a cells, exposed or not to CBX (150 µM, 48 h). **a** Representative experiment. The sequential injections were: (1) glucose 10 mM, (2) oligomycin 1 μM, (3) DNP 100 μM, and (4) a mix of rotenone 0.5 μM and antimycin A 0.5 μM. **b** KG1a cells showed a significant decrease in their ECAR values after exposure to CBX (*n* = 3). **c** CBX exposure significantly decreases the absolute production rate of mitochondrial (mitoATP) and glycolytic (glycoATP) (n=3). * indicates *P* < 0.05.

**Supplementary Figure S8.** 11β-hydroxysteroid deshydrogenase is not involved in CBX-induced antileukemic effect. **a** AML cells do not express *HSD11B1*, quantified by RT-qPCR in 22 AML cell lines. The positive control was a mix of RNA from 10 different human cell lines (Stratagene® QPCR Human Reference Total). **b** Western blot analyses confirm the lack of expression of 11β-hydroxysteroid dehydrogenase type 1 in the 6 AML cell lines (THP-1, MV4-11, KG1a, KG-1, HL-60 and Molm-13), which is not induced by exposure to CBX (150 µM, 48 h). Recombinant human protein was used as positive control. **c** The levels of cortisone and cortisol are not modified in the supernatants of AML cell lines exposed to CBX (150 µM, 48 h).

**Supplementary References**

[s1] Herault O, Hope KJ, Deneault E, Mayotte N, Chagraoui J, Wilhelm BT, *et al.* A role for GPx3 in activity of normal and leukemia stem cells. *J Exp Med* 2012;209:895–901

[s2] Vignon C, Debeissat C, Georget M-T, Bouscary D, Gyan E, Rosset P, *et al.* Flow cytometric quantification of all phases of the cell cycle and apoptosis in a two-color fluorescence plot. *PLoS One* 2013;8:e68425

[s3] Zibara K, Awada Z, Dib L, El-Saghir J, Al-Ghadban S, Ibrik A, *et al.* Anti-angiogenesis therapy and gap junction inhibition reduce MDA-MB-231 breast cancer cell invasion and metastasis in vitro and in vivo. *Sci Rep* 2015;5:12598–614

[s4] Desbourdes L, Javary J, Charbonnier T, Ishac N, Bourgeais J, Iltis A, *et al.* Alteration analysis of bone marrow mesenchymal stromal cells from de novo acute myeloid leukemia patients at diagnosis. *Stem Cells Dev* 2017;26:709–22
