# Supplementary material for: Disruption of gap junctions attenuates acute myeloid leukemia chemoresistance induced by bone marrow mesenchymal stromal cells
Source: Oncogene. 2019 Oct 24;39(6):1198–212. doi: 10.1038/s41388-019-1069-y (PMC7002301; doi:10.1038/s41388-019-1069-y)
Supplement: Supplementary file 2 — Supplemental material_Figures [file 41388_2019_1069_MOESM2_ESM.pdf]

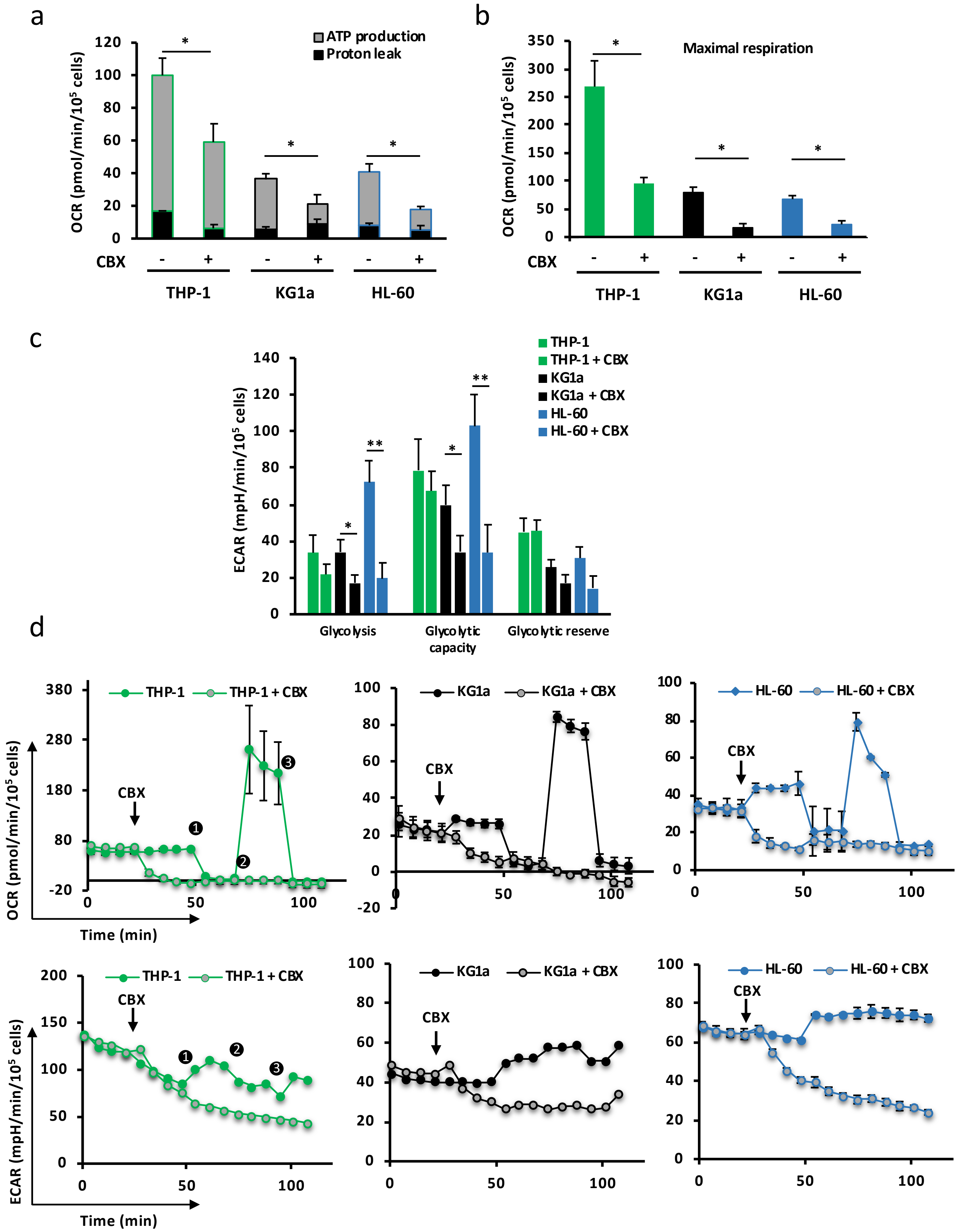

Supplementary Figure 1

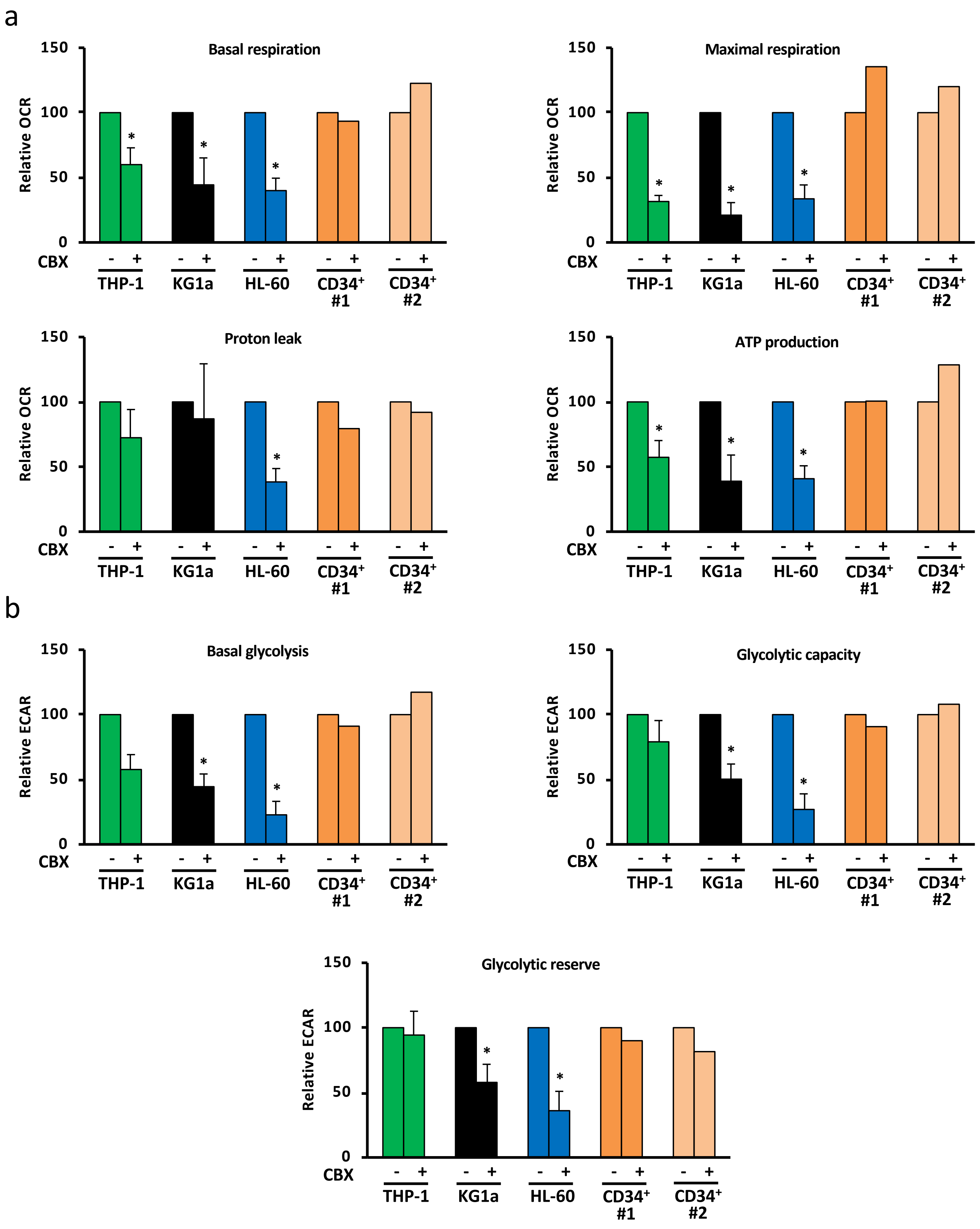

Supplementary Figure 2

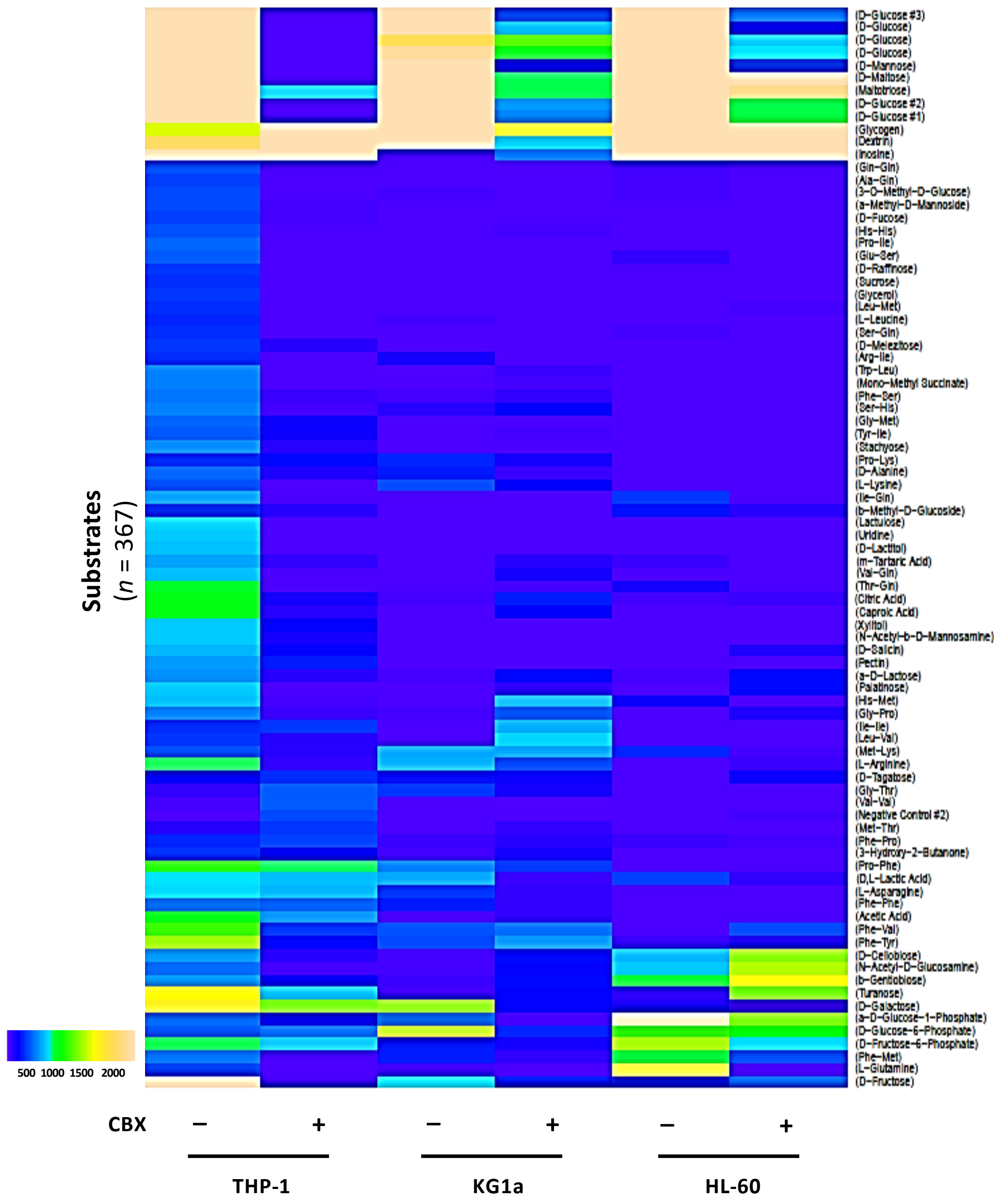

Supplementary Figure 3

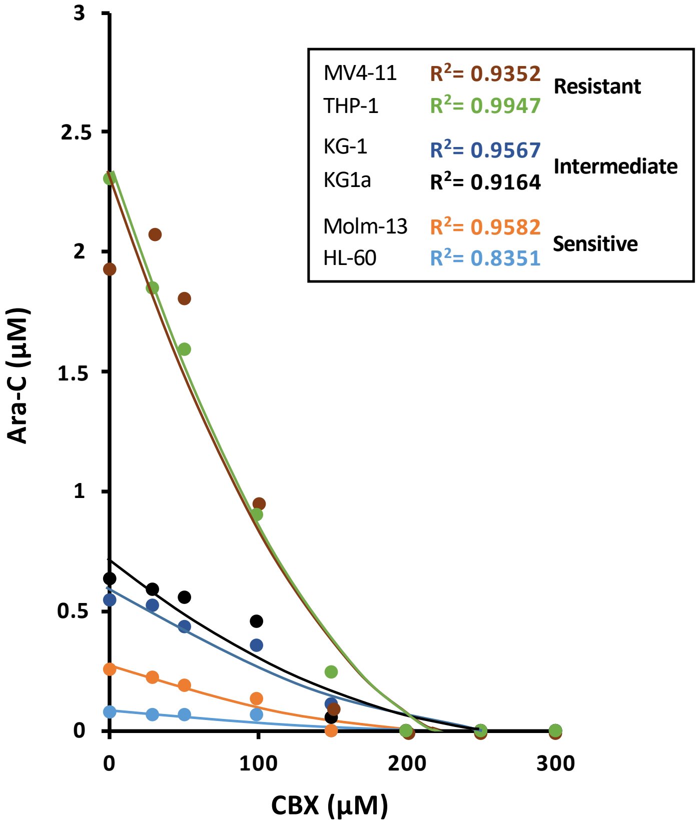

Supplementary Figure 4

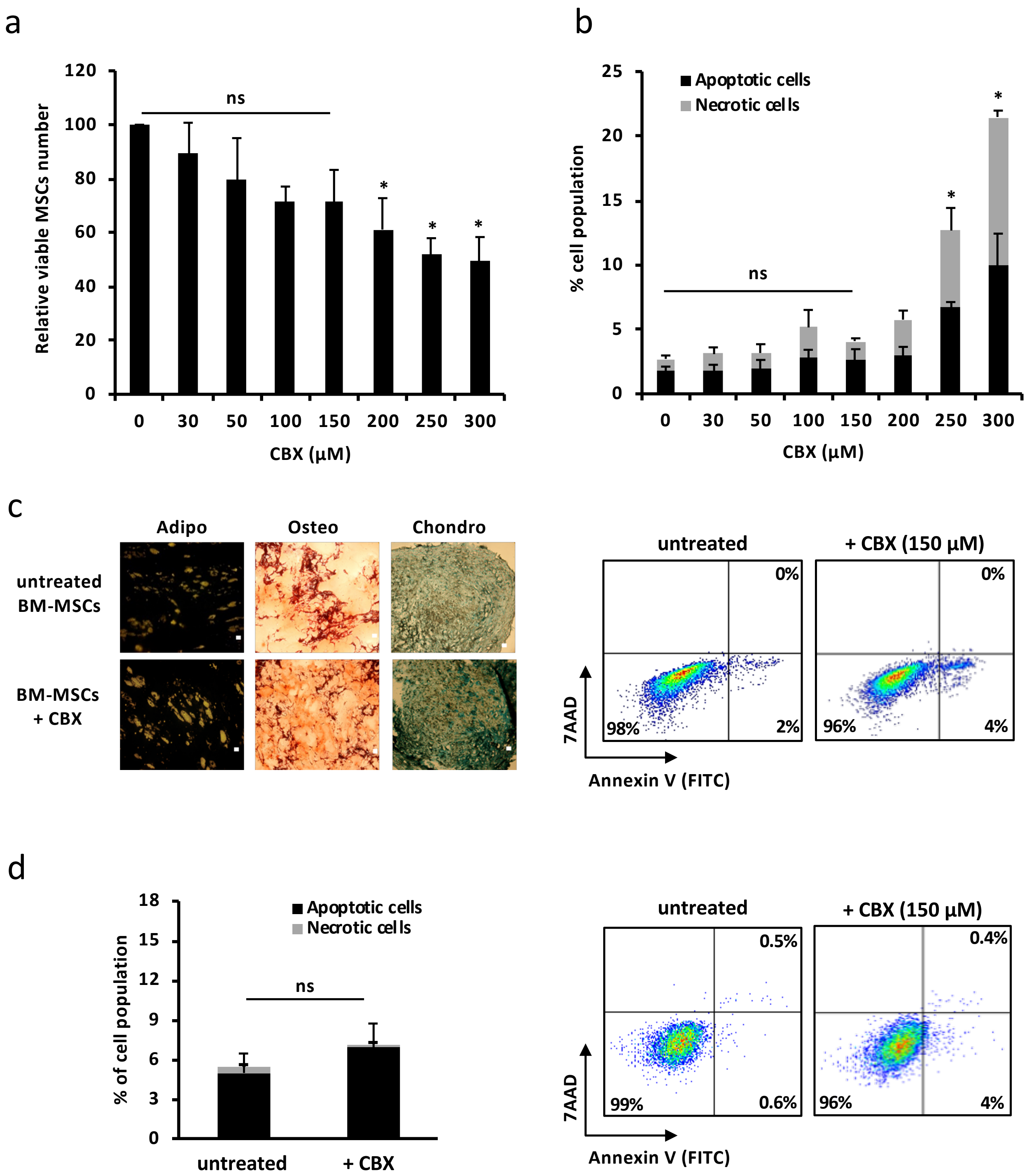

Supplementary Figure 5

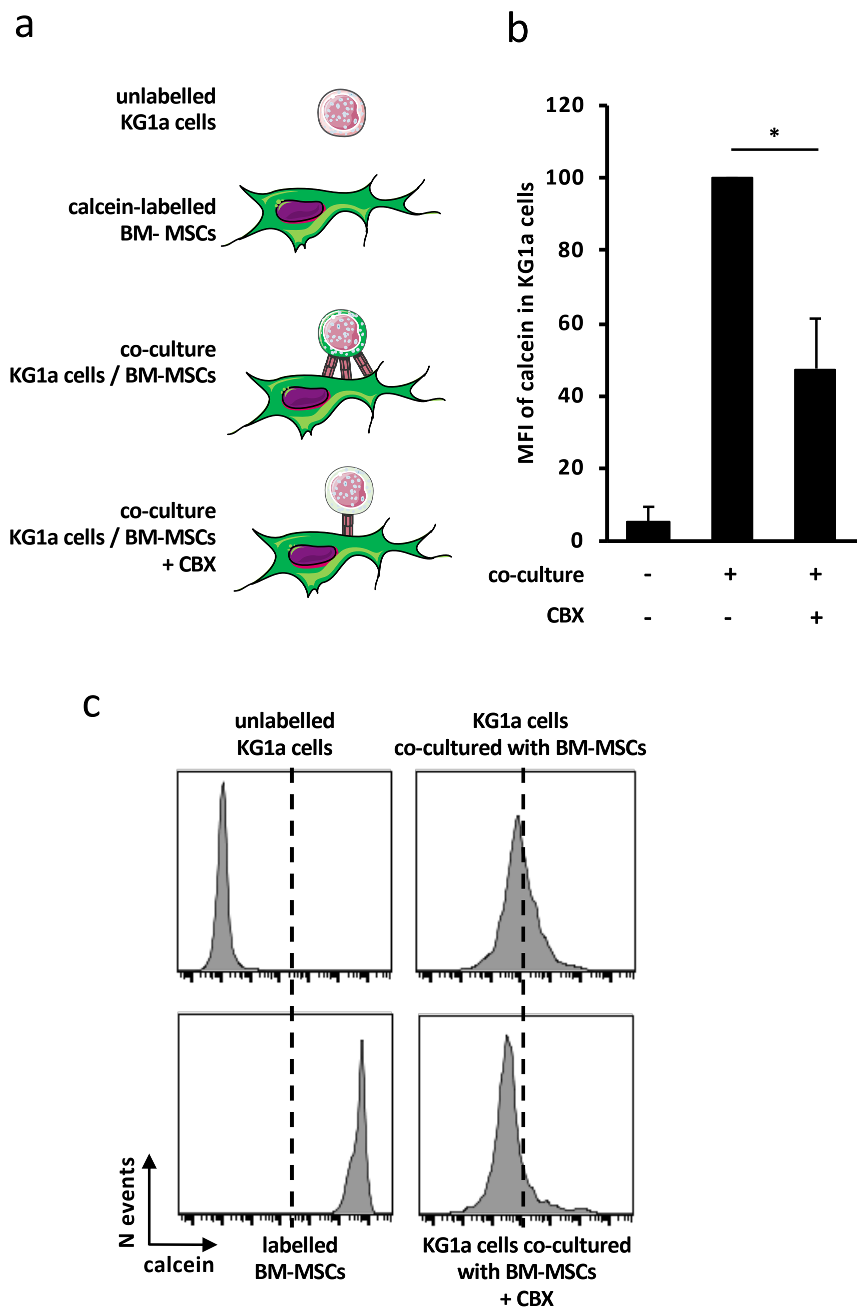

Supplementary Figure 6

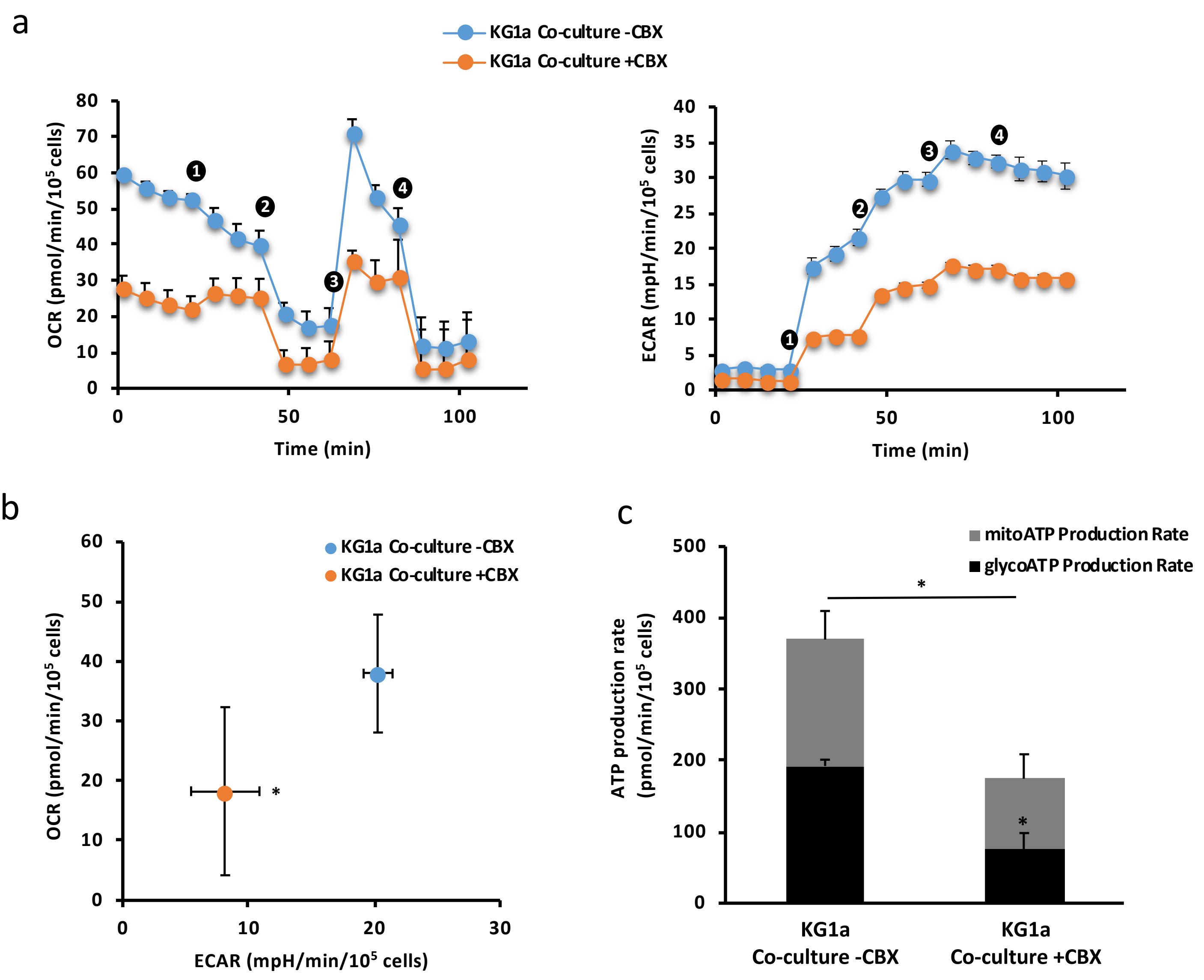

Supplementary Figure 7

a

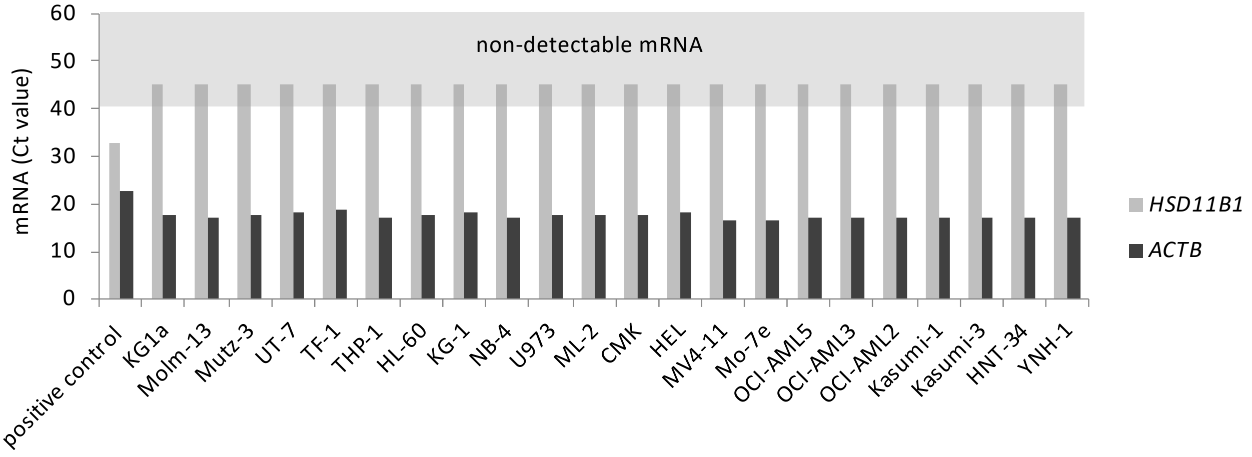

b

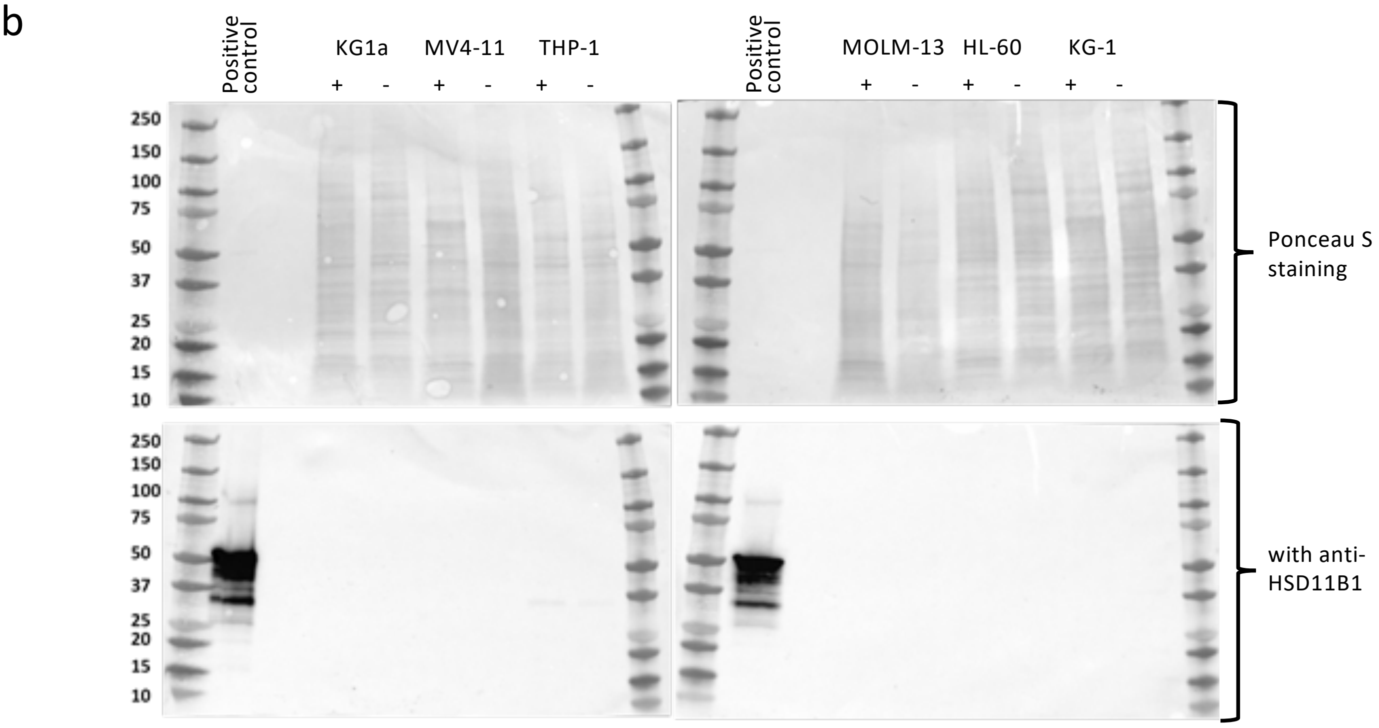

c

	cortisol	cortisone
**THP1**	0.5081	0.3576
**THP1 + CBX**	0.5064	0.3868
**MV4-11**	0.5052	0.3748
**MV4-11 + CBX**	0.5226	0.4099
**KG1a**	0.5052	0.3649
**KG1a + CBX**	0.4990	0.3996
**KG-1**	0.5097	0.3939
**KG-1 + CBX**	0.5035	0.4084
**HL60**	0.5048	0.3829
**HL60 CBX**	0.5160	0.4052
**Molm-13**	0.5286	0.3896
**Molm-13 + CBX**	0.5215	0.4106

Supplementary Figure 8
