# Supplementary material for: Disruption of gap junctions attenuates acute myeloid leukemia chemoresistance induced by bone marrow mesenchymal stromal cells
Source: Oncogene. 2019 Oct 24;39(6):1198–212. doi: 10.1038/s41388-019-1069-y (PMC7002301; doi:10.1038/s41388-019-1069-y)
Supplement: Supplementary file 3 — Supplemental material_Tables [file 41388_2019_1069_MOESM3_ESM.pdf]

Genes	Primer Sequence (Forward)	Primer Sequence (Reverse)	Probes
GJA1 (Cx43)	cgtgacttcactacttttaagcaaa	caggattcggaaaatgaaaagt	cagccaca
GJA3 (Cx46)	gccctttggaaaactgtgag	aatggctgtagttatgcaccag	tcctctcc
GJA4 (Cx37)	cctcaaaccctctcctcaca	gctctgtctgtgcgatcaag	ctgctggg
GJA5(1) (Cx40(1))	gggaggaggaaaagaagca	ttcccaggaagctccaatc	tggctgtg
GJA5(2) (Cx40(2))	caagcactgggagacgaaa	cacggtcgagtgcttgtgta	tcctccag
GJA9 (Cx59)	gaggagattggagcaagagc	ggttcctctgagtggagcttta	agctggag
GJB1(1) (Cx32(1))	cacgcctgcagacattctc	tcctgcctcattcacacct	ggcagcag
GJB1(2) (Cx32(2))	gagcaggggttggtcaag	tcacaagccacctcttcattt	ctgcctct
GJB2 (Cx26)	tgtacgacggcttctccat	cagtccacagtgttgggaca	ccagccgc
GJB3(1) (Cx31(1))	catcacttggctcagtggaa	ggaagatgggagcaccact	cctcagcc
GJB3(2) (Cx31(2))	gacatagactttgaaacaagcgaat	gctagcagtgccctaagtgg	caggcagc
GJB5 (Cx31.1)	tgcttgctgagtcctattgc	aagatactccagttcatggtgga	ctgctggg
GJB7 (Cx25)	gccaagatggctgaatagga	catcttctgcgttgctcact	cagctccc
GJC1(1) (Cx45(1))	tacagggggaaggagtcaag	cttctggcgccaaatgtc	ttcctggc
GJC1(2) (Cx45(2))	tgagtaaacatttggctctggtt	agagtcccctgagcttggat	cagcatcc
GJC3 (Cx30.2)	caaagagcaatttcaagaagca	gctcctccttggacaggatt	catcacca
GJD2 (Cx36)	tgcatcaaaatccaagctca	tttcggaacaccacttggata	cttcctgc
GJD3 (Cx31.9)	gtctagcgtctcgggttcg	agcggtgcccttagctgt	cagcatcc
GJB4 (Cx30.3)	aaacagacaagcccaaggac	caaaatcagggtctaccttattcc	ggaagcag
GJB6(1) (Cx30(1))	gggcatcctgtgttttgc	gccagatcttctgtacttcttttcc	ctgctccc
GJB6(2) (Cx30(2))	gggcatcctgtgttttgc	caatctttgctcggtcagtg	tcctgctc
GJB6(4) (Cx30(4))	gctttctgaatgtagacggaaca	tggcaaacggatgagttaaa	ggaagcag
GJA10 (Cx62)	ctggcagtgctggaaatca	gtattctgcaggccaaatgag	tgctgtcc
ACTB	attggcaatgagcggttc	cgtggatgccacaggact	gctggaag
EF1A	gaaaaatgcttttcgcgatct	tatgggaggtcaggcacag	ctgctggg
YWHAZ	gcaattactgagagacaacttgaca	ggaaggccggttaatttt	ttctcctg
RPL13A	gcaattactgagagacaacttgaca	ggaaggccggttaatttt	ttctcctg
GAPDH	agccacatcgctcagacac	gcccaatacgaccaaatcc	tggggaag
HSD11B1	ccctgtcggatggctttta	ttccttggagcatctctggt	tggcagag

Supplementary Table 1. List of primers and probes

Sex	Age	FAB subtype	% BM blasts	karyotype	Next-Generation Sequencing (NGS)
F	68.5	M1	92	46,XX, normal	NOTCH1 p.(Gln2459*); NPM1 p.(Trp288Cysfs*?); TET2 p.(Lys389Argfs*38); TET2 p.(Lys1491Argfs*80)
M	75.9	M1	92	46,XY, normal	DNMT3A p.(Ile681del); FLT3 c.1786_1837+2dup (+54pb); NPM1 p.(Trp288Cysfs*?); TET2 c.4044+1G>A; TET2 p.(Gln278*)
M	70.0	M2	75	46,XY, normal	CEBPA p.(Val314.Leu315insArgArgMet); CEBPA p.(Ala66Valfs*41); KIT p.(Asp816Val); NRAS p.(Gly13Asp); SETBP1 p.(Asn876del); TET2 p.(Gln622Argfs*17)
M	71.9	M1	91	46,XY, normal	EZH2 p.(Arg654Gly); FLT3 c.1804_1805ins48 p.(Leu601.Lys602ins16); RUNX1 p.(Arg139Profs*12)
M	70.6	M1	85	trisomy 8	ASXL1 p.(Glu1102Asp); CBL p.(Cys404Tyr); FLT3 c. 2503G>T p.(Asp835Tyr); IDH2 p.(Arg140Gln); SMC1A p.(Lys854Arg); SRSF2 p.(Pro95His)

NGS was performed on MiSeq™ system (Illumina®, San Diego, CA, USA) using a panel of 54 genes frequently mutated in myeloid malignancies (TruSight Myeloid Sequencing Panel, Illumina®): *ABL1, ASXL1, ATRX, BCOR, BCORL1, BRAF, CALR, CBL, CBLB, CBLC, CDKN2A, CEBPA, CSF3R, CUX1, DNMT3A, ETV6, EZH2, FBXW7, FLT3, GATA1, GATA2, GNAS, HRAS, IDH1, IDH2, IKZF1, JAK2, JAK3, KDM6A, KIT, KMT2A, KRAS, MPL, MYD88, NOTCH1, NPM1, NRAS, PDGFRA, PHF6, PTEN, PTPN11, RAD21, RUNX1, SETBP1, SF3B1, SMC1A, SMC3, SRSF2, STAG2, TET2, TP53, U2AF1, WT1, ZRSR2.*

Supplementary Table 2. Characteristics of primary AML
